# Interdigitated Electrode Biosensor Based on Plasma-Deposited TiO_2_ Nanoparticles for Detecting DNA

**DOI:** 10.3390/bios11070212

**Published:** 2021-06-29

**Authors:** Jhongryul Yoo, Hongin Jeong, Seo Kyung Park, Sungho Park, Je Seung Lee

**Affiliations:** 1 Department of Life Science and Chemistry, Daejin University, 1007 Hoguk Road, Pocheon-si 11159, Korea; whdfuf1345@naver.com (J.Y.); wjdghddlsdl1@naver.com (H.J.); 2 Department of Chemistry and Research Institute of Basic Sciences, Kyung Hee University, 26 Kyungheedae-ro, Dongdaemun-gu, Seoul 02447, Korea; tjrud1015@kmpc.co.kr

**Keywords:** titanium dioxide, nanoparticle, deposition, DNA, biosensor, picoammeter

## Abstract

Bioelectrodes mediated by metal oxide nanoparticles have facilitated the development of new sensors in medical diagnosis. High-purity TiO_2_ nanoparticles (NPs) were synthesized through thermal plasma and deposited directly on an interdigitated electrode. The surface of the TiO_2_-deposited electrode was activated with (3-aminopropyl) triethoxysilane (APTES) followed by fixing the single-stranded probe deoxyribonucleic acid (DNA) to fabricate the DNA biosensor. The structural properties of the deposited TiO_2_ nanoparticles were analyzed using a transmission electron microscope (TEM), X-ray diffraction (XRD), and a dynamic light scattering (DLS) system. The chemical composition and structural properties of the TiO_2_ nanoparticle layer and the fixed layer were analyzed by X-ray photoelectron spectroscopy (XPS) and scanning electron microscopy (SEM). *E. coli* O157:H7, a well-known pernicious pathogenic bacterial species, was detected as a target DNA of the prepared DNA biosensor, and the characteristics of DNA detection were determined by the current change using a picoammeter. The degree of binding between the probe DNA and the target DNA was converted into an electrical signal using the picoammeter method to quantitatively analyze the concentration of the target DNA. With the specificity experiment, it was confirmed that the biosensor was able to discriminate between nucleotides with mismatched, non-complementary, or complementary sequences.

## 1. Introduction

Due to the food poisoning epidemic that is spreading worldwide, more and more attention is being paid to public health issues. The most well-known causes of food poisoning are pathogenic bacteria including *Escherichia coli* (*E. coli*) O157:H7, *Clostridium perfringens*, *Salmonella*, *Campylobacter*, *Listeria monocytogenes*, etc. [1,2,3,4]. *E. coli* O157:H7 was first discovered in 1982 and classified as an enterohemorrhagic *E. coli* (EHEC), which is a serotype bacterial species and can produce Shiga-like toxins [5,6,7]. It is the causative agent of food-borne diseases caused by contaminated raw food, including unsterilized milk or raw ground beef [8,9]. As the incidence of bacteria-related diseases and the number of deaths continue to increase worldwide, there is an urgent need to develop a biosensor capable of quickly and inexpensively detecting pathogenic bacteria such as *E. coli* O157:H7 with high sensitivity and specificity.

Several traditional tests, such as microbial culture and microscopic counting methods, are commonly used as pathogen detection and counting methods, but have the disadvantage of being time consuming and having low accuracy. Therefore, various analytical methods have been developed including deoxyribonucleic acid (DNA) and ribonucleic acid (RNA) probe [10], micro-electromechanical systems (MEMS) [11], flow cytometry, immunomagnetic separation (IMS) [12], quartz crystal microbalance (QCM) [13], polymerase chain reaction (PCR) [14], and surface plasmon resonance (SPR) [15] to detect these organisms in food with high levels of sensitivity and specificity. Although these methods can detect bacteria faster and more sensitively than the conventional microbial culture methods, they still show false positive and false negative results, require tedious work, and have the disadvantage of being expensive for wide applications. A few other analysis methods based on chemiluminescence [16,17] and fluorescence [16] have been developed. However, these methods also have drawbacks, including being qualitative measurement methods based on labels and requiring the use of various chemical substances.

Recently, many efforts have been made to develop a portable biosensor for the detection of pathogenic bacteria which is fast, inexpensive, and has high sensitivity that can meet the needs of field testing [18]. Until now, pathogen biosensors have mainly consisted of immune detection [5,6] based on the interaction of specific antibodies and antigens and DNA-based detection [19,20,21]. However, since the immune-detecting biosensors are not easy to apply in the field due to shortcomings including the sensitivity of antibodies to changes in temperature or pH and the fact that they typically respond only to a single antigen, the research direction has shifted to the study of DNA-based biosensors that can detect specific nucleotide sequences in DNA with high specificity. A DNA biosensor able to detect single-stranded target DNA by hybridization with a complementary probe array is fabricated by the immobilization of single-stranded probe DNA having a specific sequence on the solid surface of a transducer [22,23,24]. Although many researchers have done a great deal of work on electrochemical transducers that have many advantages in DNA detection, the various chemicals used during silanization, immobilization, and hybridization processes have the potential problem of generating noise during DNA detection [19,21].

Recently, high-performance sensors based on metal nanoparticles (NPs) have been attracting attention as a promising method that can detect various target molecules. Among various NPs, a great deal of attention has recently been focused on using oxide materials because metal oxide NPs promote the redox reaction with respect to the substance to be detected, thereby changing electrical conductivity [25]. Moreover, biosensing performances can be improved by increasing the electron transfer involved in the reaction between the device and the target molecule. In addition, the immobilization of biomolecules on the device surface must be easy, and the device must be able to strongly adsorb the target material on the surface [26]. Titanium dioxide (TiO_2_) NPs, which have excellent properties such as high chemical resistance, large specific surface area, high catalytic efficiency, high electrical conductivity, and physical strength, are one of the most widely used metal oxides for sensing applications because they can improve the interaction between biomolecules and electrode surfaces [24,25,26,27,28,29,30].

Most biosensors based on conventional electrochemical detection methods using three electrodes, such as cyclic voltammetry, show poor detection limits [27]. Therefore, research on microelectronic devices that can improve sensitivity and reduce detection time is expanding. Planar interdigitated electrodes (IDEs), which consist of a pair of comb-shaped microband array electrodes and a pad at the bottom, are widely applied to the field of sensors due to their simple manufacturing process. The IDE system is very promising for biosensor application because it is capable of being label-free [28] with ultra-high sensitivity [31] and rapid detection [32], and it requires only a small amount of sample.

In this study, we fabricated a DNA biosensor using high-purity TiO_2_ nanoparticles which were deposited on an IDE using thermal plasma deposition. By measuring the change in the microcurrent flowing on the surface of the biosensor using a picoammeter, it was possible to quantitatively distinguish the target DNA with high sensitivity and specificity and to qualitatively analyze the concentration of target DNA in a sample. In particular, picoammeters provided an innovative pathway to link the DNA recognition system and signal generation, and no additional activating chemicals were required to read the small currents that changed during the interaction of DNAs. Therefore, in this study, we adopted the DC picoammeter method to develop a biosensor that can detect specific DNA easily, in real-time, without the need for a label.

## 2. Materials and Methods

### 2.1. Materials and Chemicals

TiCl_4_ (ReagentPlus^Ⓡ^, 99.9%) sealed in a bubble-type canister, (3-aminopropyl)triethoxysilane (APTES), and developer RD-6 were obtained from Sigma-Aldrich Co. (Yongin, Korea). Negative photoresist (NR) ma-N 1405 was purchased from Jaesung ITS (Anyang, Korea). Ar and O_2_ gases with high purity (99.999%) were purchased from Sinyang Co. (Seoul, Korea). The synthetic oligonucleotides of 30-base length were purchased from BioD Co. (Gwangmyeong, Korea).

### 2.2. Characterization Methods

A field-emission scanning electron microscope (FE-SEM, SU 8220, Hitachi Co., Tokyo Japan) and high-resolution transmission electron microscope (HR-TEM, Tecnai G^2^ F20, FEI Co., Hillsboro, OR, USA) equipped with an energy-dispersive spectrometer (EDS) with an EDAX PV9761 Si (Li) detector at the acceleration voltage of 200 kV were used. The presence of the NPs in the prepared films was determined by X-ray photoelectron spectroscopy (XPS) measurements with a PHI 5800 ESCA System (Physical Electronics, USA). The X-ray diffraction (XRD) patterns were recorded on an X-ray diffractometer (XRD-6000, Shimadzu Co., Japan). The currents to voltages were measured using a Keithley 6487 Picoammeter Voltage Source (Tektronix Inc., Beaverton, OR, USA) at ambient temperature.

### 2.3. Fabricating the Interdigitated Electrode and Deposition of TiO_2_ Nanoparticles

Glass substrates were cleaned with organic solvents prior to the fabrication of the interdigitated electrode (IDE) followed by drying under nitrogen atmosphere. To pattern finger electrodes in the active area, the lift-off lithography method was used. On the glass substrate, a negative photoresist (ma-N 1405) was spin-coated with a spin speed of 2000 rpm followed by heating at 150 °C for 60 s to harden the spin-coated resist layer. After developing the resist by exposing RD–6 to UV light under a photomask for 240 s, the residual solvent in the resist was removed, and the film was affixed to the substrate by heating at 100 °C for 60 s. The Pt film was deposited by a DC magnetron sputtering system (RSP 5004, SNTEK, Suwon, Korea). The photoresist was stripped using acetone followed by rinsing with distilled water until solid IDEs were exposed.

To deposit the crystalline TiO_2_ NPs, self-developed equipment capable of depositing the precursor (TiCl_4_) using thermal plasma directly on a Pt IDE patterned glass substrate was used [33]. The IDE electrode fabricated glass substrate was immobilized on the substrate holder of the nanoparticle manufacturing and deposition equipment. Ar carrier gas was injected into a bubble-type canister containing TiCl_4_ to inject a Ti precursor into a stabilized plasma flame. The resulting TiO_2_ NPs were injected into the chamber and deposited on an Pt IDE fabricated substrate.

### 2.4. Silanization of the Deposited TiO_2_ NPs

To prepare the active layer, the surface of the TiO_2_ NPs layer was functionalized with 0.02 M APTES by self-assembly inside a fume hood [34,35,36,37,38]. Next, 25 μL of APTES was dropped onto the surface of the TiO_2_-NPs-based transducer and stayed for 3 h in a drying cabinet followed by washing the unreacted APTES with deionized water (DI, > 18 MΩ). Any other chemicals for activation were not used because the picoammeter could measure the very small current changes during the immobilization of probe DNA and the hybridization of target DNA molecules.

### 2.5. Formation of the Recognition Layer on TiO_2_-NPs-Based Transducer

A carboxyl-functionalized synthetic 30-base oligonucleotide probe and 30-base target oligonucleotides were used for *E. coli* O157:H7 detection. The base sequences of the synthetic oligonucleotides probe and targets were as follows: probe oligonucleotide: (5′–(COOH) AAC GCC GAT ACC ATT ACT TAT ACC GCG ACG–3′), complementary oligonucleotide: (5′–CGT CGC GGT ATA AGT AAT GGT ATC GGC GTT–3′), noncomplementary oligonucleotide: (5′–GCA GCG CCA TAT TCA TTA CCA TAG CCG CAA–3′), and the single base mismatch oligonucleotide: (5′–CGT CGC GGT ATA ACT AAT GGT ATC GGC GTT–3′). The oligonucleotide stock solutions (10 μM) were prepared using deionized ultrapure water (>18 MΩ). The probe oligonucleotide was immobilized on the surface of the TiO_2_-NPs-deposited Pt IDE by dropping 2.0 μL of the probe oligonucleotide solution and spreading according to the previously reported method [34,35,36,37,38]. Remaining unbound probe DNA was removed by rinsing with deionized water.

### 2.6. Performance Evaluation of the Fabricated Biosensor

For this, 2.0 μL of the complementary DNA solution was dropped on the TiO_2_-NPs-deposited Pt IDE on which the probe DNA was immobilized and spread well to perform a hybridization reaction between the probe and the complementary DNA, followed by rinsing the surface with deionized water and performing the electrical measurements using a Keithley 6487 picoammeter. Other 30-base oligonucleotides were tested with the same method.

## 3. Results

**Figure 1:**
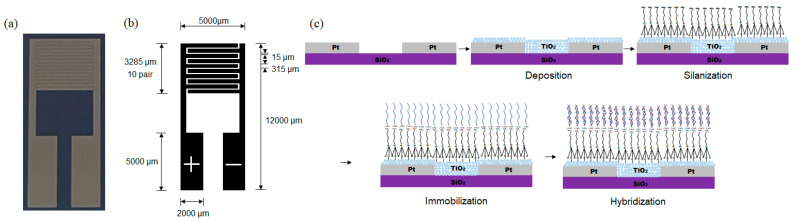
(**a**) Photograph of a Pt IDE. Schematic diagrams of (**b**) the Pt IDE with dimensions and (**c**) the fabrication process of the DNA biosensor on a Pt IDE.

Conventional electrochemical methods suffer from noise because of the use of various chemicals for obtaining the detection results. To overcome this problem, a device which detects the specific DNA without using other chemicals was developed. The Pt interdigitated electrode (IDE) was patterned on a glass substrate with negative photoresist by the lift-off lithography method instead of the conventional photolithography method. The Pt film was deposited at a pressure of ~10^−6^ Torr using a DC magnetron sputtering system, and the photoresist was stripped. Figure 1a,b shows a photograph and schematic diagram of the interdigitated electrode sensor array with dimensions 5 × 12 mm^2^. The width of the interdigitated Pt finger electrodes was fixed to 315 μm and a gap of 15 μm was used between two fingers in the active area. The size of each contact pad was 2 × 5 mm^2^.

After patterning Pt IDE on the glass substrate, the TiO_2_-DNA biosensor was fabricated in four steps as shown in Figure 1b: the thermal plasma deposition of pure TiO_2_ NPs, the silanization of TiO_2_ NPs with APTES, the immobilization of probe DNA, and the hybridization of target DNA. Single-stranded DNA (ss-DNA) was immobilized on the surface of TiO_2_ NPs through coordinated cross-linkages [39].

Pure crystalline TiO_2_ NPs were deposited directly on the Pt IDE patterned glass substrate using self-developed thermal plasma deposition equipment [33]. The injected Ti precursor (TiCl_4_) was quickly mixed with the O_2_ radical and the swirling gas to produce TiO_2_ NPs. The produced NPs were injected into the chamber and deposited on the Pt IDE fabricated substrate.

**Figure 2:**
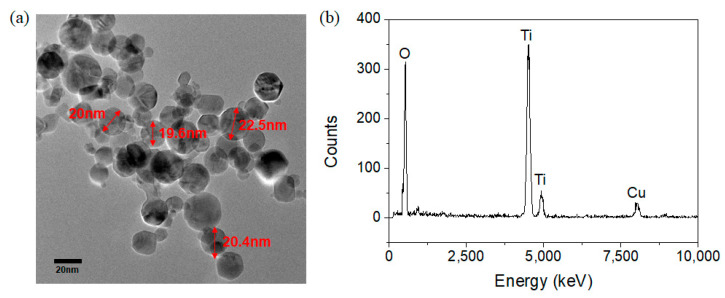
(**a**) TEM image of TiO_2_ nanoparticles synthesized by the thermal plasma process and (**b**) EDX data of TiO_2_ nanoparticles synthesized by the thermal plasma process.

A TEM image of the deposited TiO_2_ NPs by the thermal plasma process is shown in Figure 2a. The average size of the prepared TiO_2_ NPs was measured as around 20 nm. The purity of TiO_2_ NPs synthesized by the thermal plasma process was confirmed by the result of TEM-EDX observations as shown in Figure 2b.

**Figure 3:**
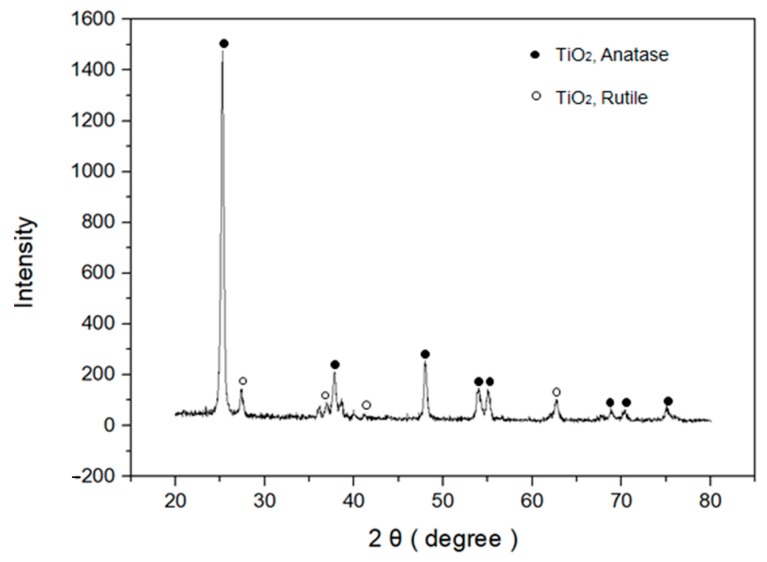
XRD pattern of deposited TiO_2_ NPs on Pt IDE.

The crystal structure of the TiO_2_ NPs deposited on the Pt IDE through the microwave thermal plasma process was analyzed by X-ray diffraction (XRD) (Figure 3). The XRD spectrum exhibited typical patterns of crystalline TiO_2_ NPs with high peak intensities, indicating that the deposited TiO_2_ NPs had a high degree of crystallinity. The peak values of the TiO_2_ XRD pattern agree well with the standard values of anatase and rutile phases (JCPDS data cards No. 21–1272 and 21–1276, respectively). The quantification of phase proportions was calculated by the method of Spurr and Myers [40], which utilizes the ratio of the rutile peak at 27.355° 2*θ* to the anatase peak at 25.176° 2*θ* as shown in Equation (1):

*W*_R_/*W*_A_ = 1.22 × (*I*_R_/*I*_A_) − 0.028 (1)

where *W*_R_, *I*_R_, *W*_A_, and *I*_A_ are the weight fractions and peak intensities of rutile and anatase, respectively. The calculated weight fraction of anatase phase (90.4%) was much higher than that of rutile phase (9.6%). This result implies that the formation of an anatase structure as a kinetic product is preferred over the formation of a rutile structure, which is thermodynamically more stable than an anatase structure due to the deposition at high temperature with short reaction time. The average size of crystalline structures can also be calculated employing the Scherer equation [40,41] as shown in Equation (2):

r = (0.9 × λ)/(B × cos*θ*) (2)

where λ, B, and 2*θ* are the wavelength of X-ray radiation, the full-width at the half-maximum of the diffraction peak, and the diffraction angle, respectively. The calculated crystallite size of deposited TiO_2_ was 22.2 nm, and this result agrees well with the TEM result (vide supra).

**Figure 4:**
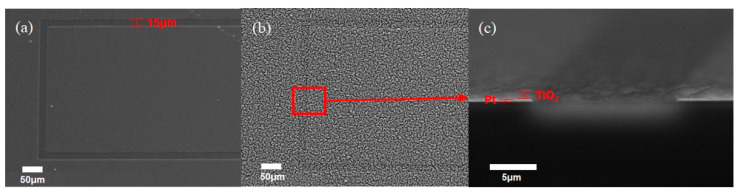
SEM image of (**a**) a bare Pt IDE, (**b**) after deposition of TiO_2_ NPs on the Pt IDE for 16 min, and (**c**) the cross-sectional image of (**b**).

SEM images of the Pt IDE are shown in Figure 4. The spacing between the finger-shaped electrodes were measured as 15 μm (Figure 4a). Figure 4b,c show the TiO_2_ NPs layer on the Pt IDE after deposition for 16 min. The TiO_2_ NPs layer was evenly deposited on the Pt IDE with a thickness of about 700 nm.

**Scheme 1:**
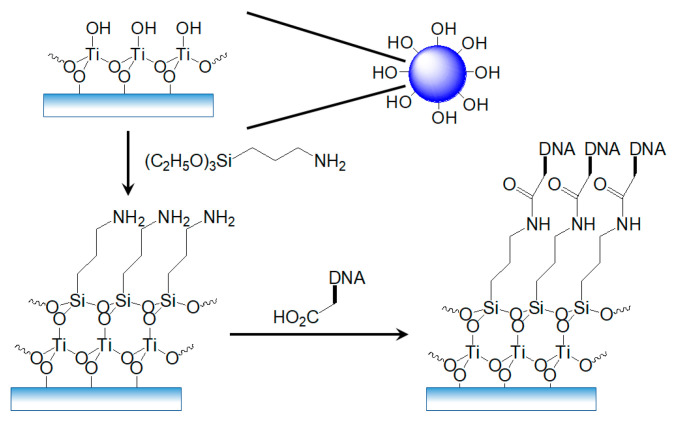
Schematic diagram of the silanization of TiO_2_ NPs and the immobilization of DNA on silanized TiO_2_ NPs.

Self-assembly of the monolayer to introduce the oligomeric DNA on the contact layer is very important for the fabrication of a DNA sensor (Scheme 1). The surface of the TiO_2_ NPs is terminated by a hydroxyl group (-OH) under atmospheric conditions, allowing the molecule to attach through a condensation reaction. For this reason, the transducer was silanized by dropping 25 μL of 0.02 M APTES solution. The aim of silanization using APTES is to modify the TiO_2_ layer by the hydrolyzation of -OH group on the surface of TiO_2_ NPs and form siloxane bonds (Si-O-Si) to immobilize the probe DNA at the surface of TiO_2_ NPs. The silane layer formed over TiO_2_ can be thin and uniform because APTES is fragile and can be easily washed away by the buffer solution [34,35,36,37,38,42].

**Figure 5:**
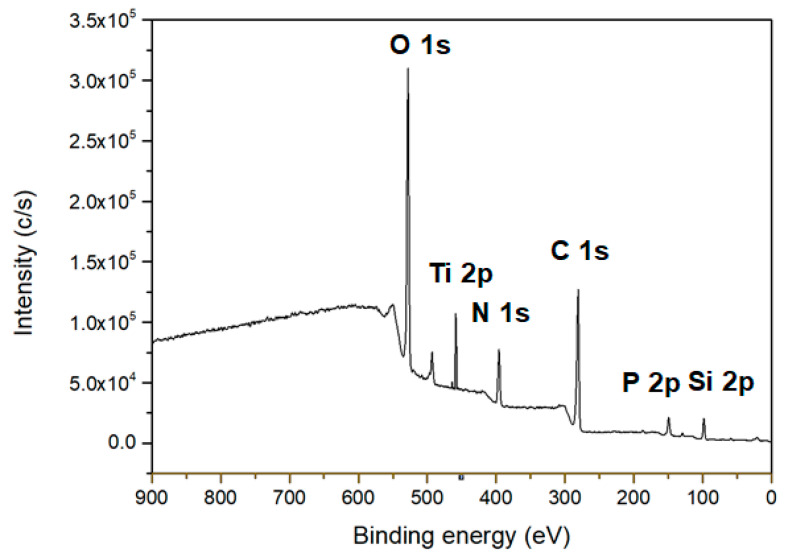
XPS survey spectra of the surface of fabricated TiO_2_-DNA biosensor.

An immobilization process of the DNA probe was carried out after silanization of the TiO_2_ NPs as shown in Scheme 1. For detection of the target DNA containing a complementary nucleotide sequence to the probe DNA, single-stranded probe DNA (ss-DNA) modified with a carboxyl group (-COOH) was immobilized on the surface of the silanized TiO_2_ NPs through the formation of an amide bond between APTES and the probe DNA. Figure 5 shows the X-ray photoelectron spectroscopy (XPS) analysis results of the electrode surface. The Ti and O are from deposited TiO_2_ NPs; the O, N, Si, C, and P are from APTES and probe DNA.

**Figure 6:**
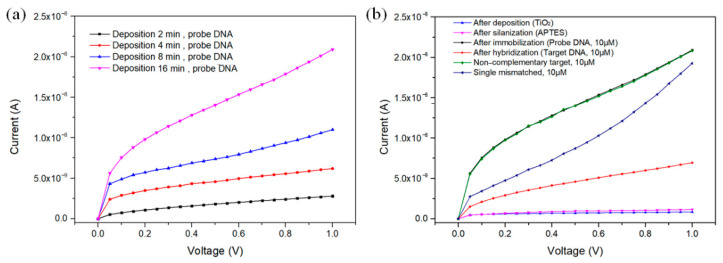
(**a**) Dependence of the TiO_2_ NPs deposition time on the responses of the sensor. (**b**) Current responses of each Pt IDE after TiO_2_ deposition, silanization, and DNA immobilization, and current responses according to the reaction between the fabricated DNA sensor and different DNA.

To monitor the performance of the developed device, instead of using the conventional electrochemical methods which have poor signal-to-noise ratios because of the use of various chemicals, the very small electric current flowing through the TiO_2_ NPs was measured with a picoammeter in a voltage range of 0 to 1 V (Figure 6). Since this method only uses deionized water for the dilution of DNA, it can greatly reduce the effects of noise generated by the various chemical substances used in conventional methods.

Figure 6 shows current–voltage curves of TiO_2_-NPs-based biosensors. In order to build a DNA sensor that does not damage the sensitive DNA molecules, the current flowing through the sensor must be very low, and quantitative detection should be possible with a high signal-to-noise ratio. At 0.5 V, as shown in Figure 6b, a very low current of 0.18 nA flowed through the sensor with only TiO_2_ NPs deposited, and only a slightly increased current of 0.36 nA flowed even after the silanization by APTES. When ss-DNA was immobilized on the sensor surface, however, the current value increased to 14.0 nA as the surface charge density was increased by DNA, which has negative charges on its backbone [43]. Figure 6a and Table 1 exhibit the current–voltage curves and average currents of the ss-DNA-immobilized sensors according to the different deposition times of TiO_2_ NPs on the surface of the Pt IDEs. As the deposition time of the TiO_2_ NPs increased, the number of TiO_2_ NPs that could immobilize the DNA molecules increased. As a result, the active surface area of the Pt IDEs increased, and accordingly, more probe DNA could be attached, and the current increased.

The selectivity of the sensor was further investigated by hybridizing three different types of DNA, each at a concentration of 10 μM individually (Figure 6b). When the ss-DNA with a complementary nucleotide sequence and the immobilized probe DNA was completely hybridized to form double-stranded DNA (ds-DNA), the current signal was greatly reduced to 4.6 nA because the negative charges became difficult to transfer to the sensor surface due to the ion blockage by the excessive negative charges of the ds-DNA. Therefore, the output current was inversely proportional to the concentration of the complementary DNA [44]. When non-complementary DNA was exposed to the probe DNA, the change in current was not observed because the hybridization had not occurred. This result shows the high selectivity of the DNA biosensor built for detection by the hybridization of DNA. Meanwhile, when the single-base-mismatched DNA was exposed to the probe DNA, the measured current was 8.6 nA. Therefore, it was confirmed that the fabricated DNA sensor using TiO_2_ NPs deposited on Pt IDE can be used as a very effective indicator which is able to distinguish abnormally mismatched oligonucleotides.

**Figure 7:**
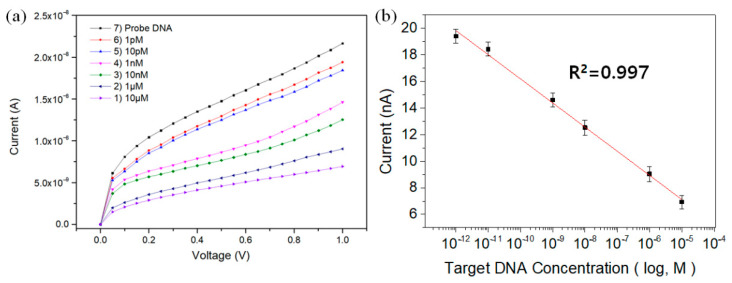
(**a**) Current responses of target DNA with varied concentrations and (**b**) calibration curve for current values at 1.0 V for varied concentrations of target DNA.

To evaluate the detection range, *E. coli* O157:H7 DNA solutions with varied concentrations were prepared and 2.0 μL of DNA solution was taken at each concentration and dropped onto the IDE sensor. Figure 7a shows the measured currents of the fabricated TiO_2_ biosensors at different *E. coli* O157:H7 DNA concentrations. The results show that there was a high correlation between the concentration of *E. coli* O157:H7 DNA and the output current. The output current of the probe DNA-immobilized IDE sensor decreased as the concentration of *E. coli* O157:H7 DNA increased. The peak current exhibited a linear relationship in the wide concentration range of 1 pM to 10 μM with the current values of 18.4 nA to 6.9 nA. The R^2^ value of the calibration curve was 0.997, indicating that the reactivity according to the target DNA concentration was excellent (Figure 7b). The sensitivity and the limit of detection (LOD) of the IDE sensor were determined as 286 nA logM^−1^ cm^−2^ and 0.43 pM (S/N = 15.5), respectively. The sensitivity was calculated using Equation (3), and LOD was obtained by applying 6 sigmas to the standard deviation value:

Sensitivity = ΔnA/(ΔlogM × sample sensing area (cm^2^)) (3)

where ΔnA is the measured current value and ΔlogM is the concentration of target DNA.

**Figure 8:**
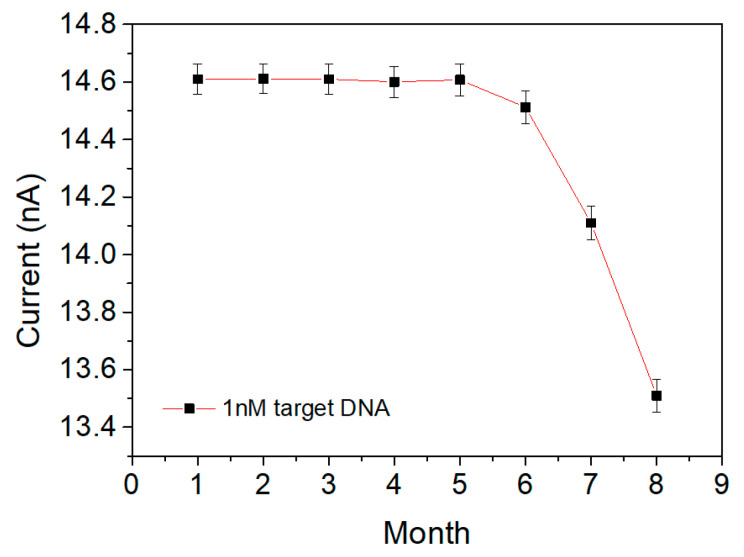
Current responses of an IDE sensor for 1 nM target DNA measured for 8 months.

Figure 8 shows the results of observing the amount of current obtained periodically for 8 months. The fabricated IDE sensor showed almost consistent responses for 6 months, and the relative standard deviation (RSD) was calculated as 3.9%. After that, a decrease in current amount of about 7.5% was observed for 2 months. This is a similar result to the previous report in which current responses decreased due to the stacking interaction of base pairs present in the probe DNA immobilized on the sensor surface [43].

## 4. Conclusions

A high-purity TiO_2_-NPs-based microchip biosensor array plasma-deposited on Pt IDE for the detection of *E. coli* O157:H7 DNA hybridization was successfully fabricated. The biosensor was fabricated by synthesizing TiO_2_ nanoparticles in thermal plasma, depositing them directly onto a Pt IDE, and then silanizing them to combine single-stranded DNA with complementary nucleotide sequences with target DNA. The fabricated biosensor was able to detect target DNA with high sensitivity and selectivity using a picoammeter. This picoammeter biosensor is distinguished from conventional electrochemical methods by its simple yet low-noise biosensing method that does not require any chemicals other than DI water. In addition, for the production of TiO_2_ NPs, it was possible to obtain high sensitivity compared to a conventional biosensor by using direct deposition using a plasma process instead of the conventional sol–gel method. For this reason, we judge that the purity of TiO_2_ nanoparticles deposited by the plasma process is higher than that of those produced by the sol–gel method, and the surface area is wider. The results of this study are expected to be utilized in the development of a field diagnostic biosensor that can diagnose high-risk infectious diseases such as foot-and-mouth disease and African swine fever without complicated pre-treatment and cloning procedures in the field.

## Figures and Tables

**Table 1:** Average currents of DNA-immobilized sensors with varied TiO_2_ NPs deposition time.

Deposition Time (min)	Average Current (nA)	
	0.5 V	1.0 V
2	1.8	2.8
4	4.5	6.2
8	7.4	11.0
16	14.0	20.9

## Data Availability

Not applicable.
